# A Non-Verbal Turing Test: Differentiating Mind from Machine in Gaze-Based Social Interaction

**DOI:** 10.1371/journal.pone.0027591

**Published:** 2011-11-09

**Authors:** Ulrich J. Pfeiffer, Bert Timmermans, Gary Bente, Kai Vogeley, Leonhard Schilbach

**Affiliations:** 1 Neuroimaging Group, Department of Psychiatry and Psychotherapy, University Hospital Cologne, Cologne, Germany; 2 Department of Media and Social Psychology, Faculty of Human Sciences, University of Cologne, Cologne, Germany; 3 Institute of Neuroscience and Medicine – Cognitive Neurology (INM3), Research Center Jülich, Jülich, Germany; 4 Max-Planck-Institute for Neurological Research, Cologne, Germany; University of Regensburg, Germany

## Abstract

In social interaction, gaze behavior provides important signals that have a significant impact on our perception of others. Previous investigations, however, have relied on paradigms in which participants are passive observers of other persons’ gazes and do not adjust their gaze behavior as is the case in real-life social encounters. We used an interactive eye-tracking paradigm that allows participants to interact with an anthropomorphic virtual character whose gaze behavior is responsive to where the participant looks on the stimulus screen in real time. The character’s gaze reactions were systematically varied along a continuum from a maximal probability of gaze aversion to a maximal probability of gaze-following during brief interactions, thereby varying contingency and congruency of the reactions. We investigated how these variations influenced whether participants believed that the character was controlled by another person (i.e., a confederate) or a computer program. In a series of experiments, the human confederate was either introduced as *naïve* to the task, *cooperative*, or *competitive*. Results demonstrate that the ascription of humanness increases with higher congruency of gaze reactions when participants are interacting with a naïve partner. In contrast, humanness ascription is driven by the degree of contingency irrespective of congruency when the confederate was introduced as cooperative. Conversely, during interaction with a competitive confederate, judgments were neither based on congruency nor on contingency. These results offer important insights into what renders the experience of an interaction truly social: Humans appear to have a default expectation of reciprocation that can be influenced drastically by the presumed disposition of the interactor to either cooperate or compete.

## Introduction

In the last decades, considerable knowledge has been acquired about how we perceive other persons, how we interpret their non-verbal behavior, and how we ‘read’ their minds. However, most experimental paradigms used to this end have relied on testing individuals in isolation. Thus, social interaction is investigated *without* interaction (‘offline’ social cognition), seemingly reflecting the view that social cognition can be sufficiently understood by investigating what a single person thinks or believes [1]. In recent years, this cognitivist and individualist approach to social cognition has been subject to criticism as it fails to incorporate the interaction process in itself, i.e. the embodiment of agents in an interaction, and the situated nature of social interaction (‘online’ social cognition, [2]). Instead, enactive accounts of social cognition have gained popularity and suggest to investigate interaction partners in true dyadic interactions [1], [3]–[5]. These accounts are based on the propositions that i) perception and action are inseparable from each other, and that ii) meaning emerges from the active exploration of and coupling with the environment.

One major reason for the scarcity of truly interactive studies in social cognition research might be the complexity of studying complex social interaction processes involving the exchange of subtle and transient cues under standardized laboratory conditions. However rich everyday social interactions present themselves, it is of great importance that the bandwidth of the interaction is restricted substantially in order to study core processes of interaction whilst maintaining acceptable levels of experimental control. Keeping this in mind, any endeavor of assessing real social interaction in fact faces two major challenges. First, an experimentally controllable domain of social cues needs to be identified. Second, a task that reliably separates and contrasts social and non-social interaction must be established.

The first challenge can be met by starting from a subset of communicative cues, which have high explanatory value for social cognitive processes and exchange in social encounters and are at the same time objectively measurable and controllable in an experimental setting. Such a cue system is ideally represented by human gaze. Gaze behavior has long been demonstrated to provide a highly informative window into social cognition [6], [7]. Here, an important aspect of social interaction is the ability to follow another person’s gaze and share a perceptual experience with someone else, thereby engaging in triadic relations between self, other, and the environment in joint attention [8]. Joint attention is believed to be crucial for an understanding of other minds [9]. An essential distinction has been made with respect to the person who is initiating joint attention and who is responding to bids of joint attention [10]. In line with observations from non-typically developing humans and research in non-human species, Moll and Tomasello [11] argue that the natural motivation to engage others in triadic interactions represents a uniquely human cognitive factor which might ultimately foster the development of a shared reality [12]. In addition, as the act of looking is both a source of stimulation and a response, perception and action are inseparable in this channel of non-verbal behavior and can hence be subject to tight experimental control [13].

A powerful paradigm to analyze social gaze in a truly interactive way has been introduced recently [14] using interactive eye-tracking and gaze-contingent eye movement simulation. This setup allows to track a person’s gaze on a stimulus screen and to control the gaze behavior of an anthropomorphic virtual character [15] dependent on the current gaze position. For the first time, this permits the exploration of gaze-based social interaction in an experimentally controllable way. In an initial study employing this interactive eye-tracking setup in a functional magnetic resonance imaging (fMRI) environment, it could be shown that self-initiated joint attention, i.e. making the virtual character follow one’s own gaze, recruits reward-related neurocircuitry consistent with the above described idea of an intrinsic motivation to jointly attend to aspects of the environment [16].

Based on this paradigm, we have developed a gaze-based version of what is known as the “Turing test” in order to study which parameters of gaze-based interactions influence humanness ratings of the virtual character. The Turing test was proposed by the British mathematician Alan Turing in order to address the question whether machines can think, i.e., whether or under which circumstances humans would ascribe human-like intelligence to machines. In order to address this question he suggested various experiments, one of which later became known as the standard Turing test. In this test, a human participant engages in verbal conversation via a computer screen with another human and a computer placed in separate rooms via a computer screen and has to judge with whom he is interacting [17]. If the participant cannot reliably distinguish between the human and the computer conversation partner, the machine is said to have passed the test. The rationale of this paradigm was used in our study to investigate humanness ascriptions during interaction.

**Figure 1:**
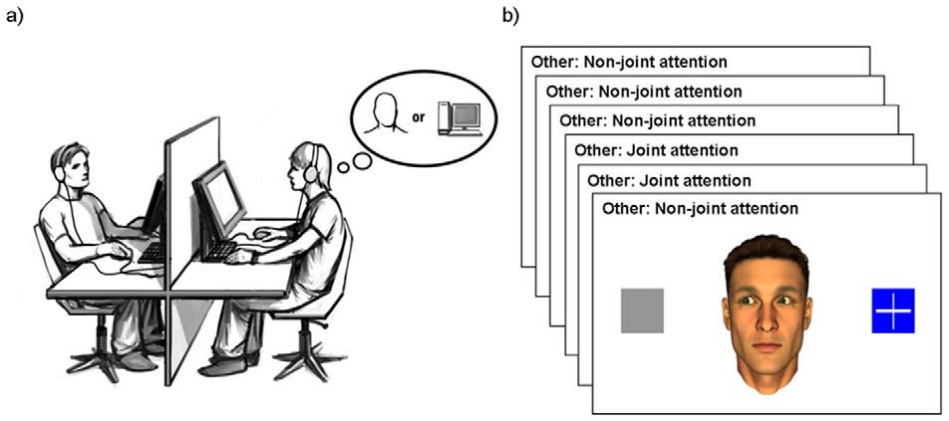
The non-verbal Turing test. *(a)* Set-up of the experiment with a volunteer participating in the study on the right and a confederate of the experimenter acting as a putative interaction partner on the left. *(b)* One exemplar interaction block of the experiment consisting of six interaction events.

For this purpose, we created a gaze-based version of the Turing Test, which in the following will be referred to as the “non-verbal Turing test”. In this test participants engage in the ascription of human agency during social interaction, which will be referred to as “ascription of humanness” throughout this article. They have to judge whether they interact with a real human or a computer based on the gaze behavior displayed by an anthropomorphic virtual character in response to their own gaze behavior (see Fig. 1a), while in fact the latter is always the case and the putative other participant is a confederate of the experimenter. Each interaction between participant and agent consisted of six events, during each of which the virtual character would either follow the participant’s gaze toward an object that was also shown on the screen or look away from that object (see Fig. 1b). The experimental manipulation consisted in the systematic variation of the number of gaze-following reactions from zero (i.e. character always looking in the opposite direction) to six (i.e. character always following) out of six possible times. In a between-subject design, we also addressed the influence of prior knowledge about the putative interactor's behavioral predisposition in order to model different social contexts. To this end, we introduced the interactor as either *naïve* to the task, *cooperative*, or *competitive*.

**Figure 2:**
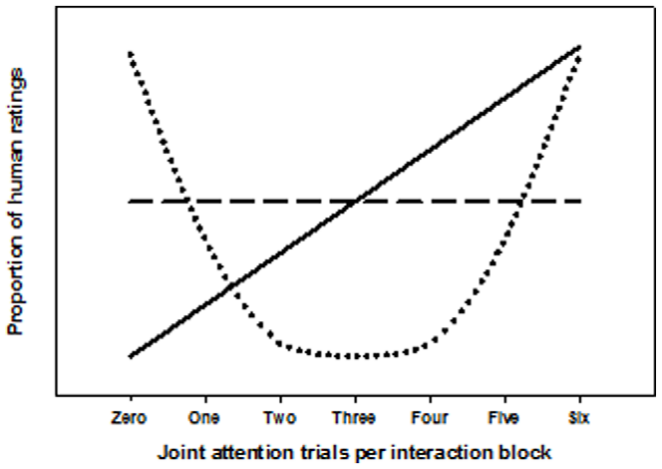
Hypotheses of humanness ascription under changing situational demands are depicted here as simple models. (1) Naive interaction: The ascription of humanness is based on maximally congruent reactions (solid line). (2) Cooperative interaction: The ascription of humanness is based on the mere contingency of reactions (dotted line). (3) Competitive interaction: The ascription of humanness is neither based on congruency nor on contingency of gaze reactions (dashed line).

Based on the literature we hypothesized three distinct outcomes in the different conditions: *(1) Congruency-based judgment in naïve interaction:* The significance of self-initiated joint attention in social cognition has been highlighted above. Particularly the data by Schilbach et al [16] suggest a motivational aspect of initiating joint attention that is reflected both on the neural and the behavioral level. This might be taken to suggest that humanness ascription should increase with increasing congruency of gaze behavior, i.e. that the experience of interacting with another person increases with the degree of gaze-following when nothing else is known about this person. *(2) Contingency-based judgment in cooperative interaction:* In definitions of cooperation, particular emphasis is put on the necessity of coordination between the cooperative interactors [18]. Therefore, we hypothesize that any form of coordinated reactions could be taken as indicative of a human interaction partner. Importantly, not only maximal gaze-following but also maximal gaze aversion is a highly coordinated interaction pattern as both patterns are maximally contingent upon the participant’s gaze. The difference with respect to the participant’s gaze is that one pattern is congruent and the other is incongruent. Hence, if coordination played a greater role in humanness ascription when encountering a cooperative interactor, contingent rather than merely congruent reactions should inform participant’s judgments. *(3) Unpredictability-based judgment in competitive interaction:* In the light of the hypotheses on how humanness is ascribed in situations with a naïve or a cooperative interactor, it might be anticipated that participants would expect a competitive person to avoid any patterned response and hence will not interpret any form of congruency or contingency as indicative of a competitive interactor. Figure 2 provides an illustration of these hypotheses.

## Methods

### Participants

In total, 128 healthy male and female volunteers aged 19 to 42 years (mean age  = 26.72±5.31), with no record of neurologic or psychiatric illnesses participated in the study. They were recruited using an internet-based system [19]. All participants were naïve with respect to the task and to the scientific purpose of the study and were equally compensated for their participation (10 Euro/hour). In the beginning of the study participants were asked to sign a written consent form in which they approved that participation is voluntary and that data are used in an anonymized fashion for statistical analysis and scientific publication. The study strictly followed the WMA Declaration of Helsinki (Ethical Principles for Medical Research Involving Human Subjects) and was presented to and approved by the ethics committee of the medical faculty of the University of Cologne, Germany.

### Setup and Materials

We made use of a recently developed interactive eye-tracking paradigm [14]. This method allows participants to interact with an anthropomorphic virtual character by means of their eye-movements. In order to detect participants’ eye-movements we used a high resolution eye-tracking system with a digitization rate of 50 Hz and an accuracy of 0.5° (TobiiTM T1750 Eye-Tracker, Tobii Technology AB, Sweden). Participants were seated at a distance of 80 cm in front of the device. Stimuli were presented on the 17” TFT screen of the eye-tracking device with screen resolution set to 1024 by 768 pixels. The viewing angle was 32×24 degrees for the whole screen. A PC with a dual-core processor and a GeForce 2 MX graphics board controlled the output of the eye-tracker as well as stimulus presentation at a frame rate of 100 Hz. Via a fast network connection gaze position updates were transferred to dedicated gaze extraction software (ClearviewTM, Tobii Technology AB, Sweden) which produced real-time gaze position output. This was made available to and used by the Presentation software (PresentationTM, http://www.neurobs.com) to control stimuli in a gaze-contingent manner.

### Task

The interaction was organized in interaction blocks of six events each (Fig. 2b). Each of these events had the following order: Participants first had to look at the virtual character. Once the program had detected a fixation of the virtual character two grey squares appeared on the left and the right side of the screen (see [14] for details on the gaze processing algorithm). Participants subsequently had to choose one of the squares by fixating it. Upon fixation the chosen square changed its color from grey to blue to provide feedback about successful gaze detection for the participant. Participants were told that their first gaze to one of the squares (but not the color change) was transferred to the screen of the eye-tracking device of the other participant in real time and that they would see the other participant’s response to this as visualized by the eyes of the virtual character visible on their stimulus screen.

As part of the “cover story”, participants were told that in a given interaction block the eye-movements of the virtual character could either be controlled by the partner or by a computer program. After each block, the participant’s task was to judge whether they had been interacting with the human partner or with the computer program. In actual fact, the other person was a confederate of the experimenter and the eye-movements of the virtual character were always controlled by the computer algorithm. Interaction blocks consisted of six interaction trials, thus allowing for a systematic manipulation of the virtual character’s gaze-following or gaze aversion behavior from zero to six out of six (0/6 to 6/6) possible times. Gaze-following thereby constituted a joint attention event, whereas gaze aversion constituted a non-joint attention event. Overall, this resulted in seven conditions (0/6, 1/6, 2/6, 3/6, 4/6, 5/6, 6/6) each of which was repeated eight times in a fully randomized fashion during the course of the experiment. The latency of the virtual character’s gaze reaction was jittered between 350 and 600 milliseconds. This resulted in gaze latencies that have previously been found to appear natural to participants (unpublished data). Joint and non-joint attention events were distributed randomly within each interaction block. At the end of each block participants were asked to indicate via button press whether they had been interacting with the other person or the computer program.

### Procedure

At the beginning, participants were seated at a distance of about 80 cm from the eye-tracking device. Instructions were provided in a standardized manner on the screen. Participants were informed that during the experiment they would be asked to engage in interaction with a virtual character presented on a computer screen in front of them by looking at the character and by looking at objects also visible on the screen. After the participant was briefed (see descriptions of experiments 1 – 5 for details), the confederate (in the following referred to as the “interactor”), who was said to be instructed simultaneously by a second experimenter in a different room, was brought into the testing room and seated in front of the second eye-tracking device. The two persons were placed about 4 meters apart from each other and were visually separated by a room-divider. The experimenter then engaged in a brief, scripted conversation with the interactor, thereby repeating some of the instructions to make the cover story believable for the actual participant. Before the experiment started, the participants’ sitting position in front of the eye-tracker was optimized and the eye-tracker was calibrated using a five-point calibration routine to obtain valid gaze positions in a stimulus-related coordinate system. The participant was lead to believe that exactly the same procedure was applied for the interactor. Subsequently, the real participant engaged in three interaction blocks to be familiarized with the task. After this practice session, remaining questions of the participant were answered. Both the participant and the interaction partner were then instructed not to communicate verbally with each other during the experiment and were asked to wear headphones in order “to prevent acoustical interferences” with their task performance. The eye-trackers were then recalibrated and the experiment started. After 28 of the 56 interaction blocks there was a 30 second break. Upon completion of the experiment, the partner was brought to another room while the participant was asked to fill out a brief questionnaire in which they had to indicate how difficult they had found the task on a 4-point scale, whether they had based their decision on considerations of human behavior or computers, whether they had used a certain strategy in the interaction, and whether there were specific criteria on which they based their decision. They were also asked to explicitly describe potential strategies and criteria. After completion of this questionnaire, all participants were debriefed and informed about the goal and purpose of the experiment. In total, the complete experimental session lasted approximately 50 minutes.

### Data Analysis and Presentation

All data were analyzed using PASW Statistics 18 (SPSS Inc, Chicago, IL, www.spss.com). One-way ANOVAs for repeated measures were used to analyze the effect of the degree of gaze-following which was included in the analysis as a factor with seven levels. In order to be able to apply parametric statistics on proportional data, such as obtained from participant’s judgments, all data were arcsine transformed [20], [21]. Planned polynomial contrasts were applied for trend analysis. In addition to the main manipulation of the task, i.e. the systematic variation of the virtual character’s gaze-following behavior, the gaze behavior of the participants themselves was analyzed to detect possible influences on the ascription of humanness. Whenever appropriate, i.e. for main effects and planned contrasts, omega squared (ω^2^) is reported as a measure of effect size [22]. The following conventions for interpreting ω^2^ are suggested: Small effects: ω^2^<0.06; Moderate effects: ω^2^>0.06 and ω^2^<0.15; Large effects: ω^2^>0.15 [23]. In the graphs representing the data, non-transformed data are used with error bars indicating the 95% confidence intervals. Post-experiment debriefing questionnaires were analyzed by an independent rater blind to the conditions of the study.

## Results

### Gaze Behavior of Participants

Before assessing the ascription of humanness based on the gaze reactions of the virtual character, we aimed at excluding potential effects of participants’ own gaze behavior on performance. Two aspects of participants’ gaze behavior were evaluated. In a first step, we investigated whether participants looked equally often to the left and right objects across conditions. This was clearly the case as indicated by left/right-ratios (Exp.1: 1.08, Exp.2: 1.04, Exp.3: 1.1, Exp.4: 1.04, Exp.5: 1.08) and supported by a one-way ANOVA comparing these ratios across conditions that did not yield any significant differences, *F*(4, 108)  = 2.08, *p* = .10. In addition, the consistency of participants’ gaze behavior was also taken into account. This is important because it is conceivable that whereas some participants alternate randomly between the left and right objects throughout interaction blocks, others chose to always fixate one of the two objects, thereby expressing higher consistency in their behavior. To assess the possibility that differences in consistency influence how participants experience the virtual character’s gaze reactions and thus possibly their humanness rating, the longest chain of consecutive gaze shifts to the same object was extracted from each interaction block and used to calculate an average consistency index for each participant and each condition. An ANOVA comparing the average consistency across experiments did not yield any significant differences, *F*(12, 408)  = 1.11, *p* = 0.35. Subsequently, the humanness ratings of each condition with the consistency index of that condition were correlated. The Pearson correlation coefficients were then included as a covariate in the repeated-measures ANOVAS employed for the within-group analyses of the effect of the independent variable (i.e. character's gaze-following behavior) on the dependent variable (i.e. the ascription of humanness) that will be described in the following sections.

### Experiment 1: Interaction with a Naïve Confederate

In what we consider as the baseline task, the confederate was introduced as naïve to the participants’ task. This means that he did not know that the real participant had to answer the question whether he had the impression to be interacting with another human or a computer program. We explicitly instructed participants that the confederate was unaware of the computer program randomly taking control of the virtual character’s eye movements and of their task and thus could not knowingly help them in answering the question.

#### Participants

26 healthy volunteers participated in this study (M = 26.34, SD = 5.12; 14 female). One female and one male participant needed to be excluded from the analysis due to technical problems during the experiment.

#### Results

**Figure 3:**
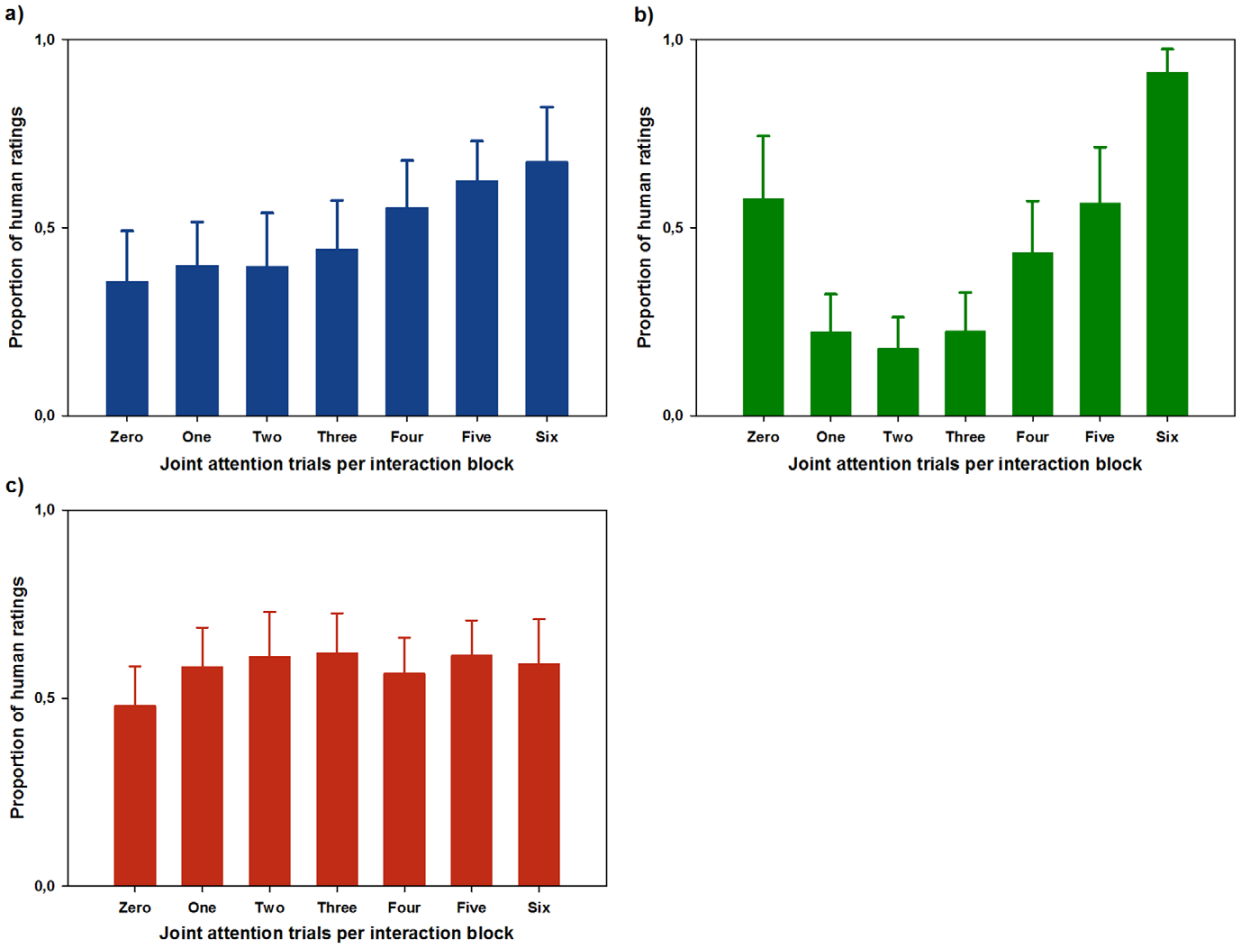
Experiments 1, 2, and 3: The ascription of humanness to a virtual character during interaction with an interactor that is *(a)* supposedly naïve to the participants’ task, *(b)* introduced as cooperative, *(c)* or as competitive.

The effects of increasing degrees of gaze-following on humanness ascription are depicted in Figure 3a. The results indicate that the proportion of human ratings increases with an increasing degree of gaze-following by the virtual character. A one-way repeated measures ANOVA including the degree of gaze-following as a factor with seven levels was performed on the data. Mauchly’s test indicated that the assumption of sphericity was violated (χ^2^ = 59.83, *p*<.001). Degrees of freedom were therefore corrected by using the Greenhouse-Geisser estimates of sphericity (ε = .54). The results show a main effect of gaze-following on the ascription of humanness, *F*(3.23, 74.34)  = 5.31, *p* = .002, ω^2^ = 0.12. Polynomial contrasts revealed a significant linear trend, *F*(1, 24)  = 13.54, *p* = .001, ω^2^ = 0.26, thereby confirming the initial observation.

#### Discussion

Consistent with the literature on social gaze and social interaction, we hypothesized that participants would base their decision on congruent reactions to their own behavior. Indeed, the results show a highly significant linear trend and demonstrate that, when interacting with a putatively naïve confederate, participants’ ratings in favor of a human interaction partner increased with increasing degrees of gaze-following. This indicates that during interaction with an unknown person there might be a default expectation of congruent reactions.

### Experiment 2: Interaction with a Cooperative Interaction Partner

It has been argued that humans have a predisposition to interact cooperatively as soon as they interact [24], [25]. To assess whether the introduction of an explicitly cooperative context would either reinforce the congruency-based pattern of humanness ascription found in the previous experiment or would rather lead to a contingency-based pattern, we introduced the interaction partner as being aware of the participants’ task in experiment 2. In addition, he was described as having been instructed to “cooperate”, thus making the task as easy for the participant as possible. To stimulate a cooperative mindset, we also informed the participant that they both would receive additionally money if cooperation would lead to more correct decisions between human interactor and computer program.

#### Participants

28 volunteers participated in this experiment (M = 26.96, SD = 6.65; 13 female). Two male participants were excluded because they did not believe the cover story.

#### Results

Figure 3b illustrates the mean responses for participants interacting with an interactor previously introduced as cooperative. Mean responses provide a first hint that during cooperative interaction the mere contingency seems to play an important role in humanness ascription. Again, Mauchly’s test showed that the assumption of sphericity was violated (χ^2^ = 80.92, *p*<.001), and the Greenhouse-Geisser correction was used (ε = .40). Here, too, the degree of gaze-following had a highly significant effect on the ascription of humanness, *F*(2.37, 59.3)  = 22.63; *p*<.001, ω^2^ = 0.38. There were highly significant linear, *F*(1, 25)  = 20.48; *p*<.001, ω^2^ = 0.20, quadratic, *F*(1, 25)  = 38.3; *p*<.001, ω^2^ = 0.47, and cubic, *F*(1, 25)  = 9.2; *p* = .005, ω^2^ = 0.05, trends describing the u-shaped response pattern. A repeated-measures ANOVA including cooperativeness (experiment 1 vs. experiment 2) as a between-subjects factor showed that there was a significant difference in humanness ascription between experiments 1 and 2, *F*(3.13, 150.17)  = 7.04; *p*<.001.

#### Discussion

Introducing the putative interaction partner as cooperative had a striking influence on the pattern of the ascription of humanness to the virtual character, which primarily followed a contingency-based pattern. Participants appear to discount the expectation of congruency of an interactor’s reaction if the interactor is introduced as cooperative, indicating that in a cooperative context coordinated reactions seem to be more indicative of a human interactor than simple congruent reciprocation.

### Experiment 3: Interaction with a Competitive Interaction Partner

This experiment assessed whether one of the prevalent response patterns from experiments 1 and 2 would still appear in a competitive situation. To this end, participants were informed that the confederate was aware of their task and instructed that he should behave in a competitive way, hence making the decision as difficult as possible. To accentuate this manipulation, participants were told that they could earn extra amounts of money depending on their success rate. Conversely, the reimbursement of the other person was said to depend on his ability to trick the participant. It was hypothesized that participants would avoid the ascription of humanness in situations of maximal congruency or contingency of gaze reactions.

#### Participants

21 healthy volunteers participated in this experiment (M = 29.9, SD = 4.95; 9 female).

#### Results

In Figure 3c the ascription of humanness in the presence of a competitive interactor is depicted. It is obvious that none of the previously described response patterns can be observed. Again, the assumption of sphericity was violated (χ^2^ = 35.35, *p* = .02) and the Greenhouse-Geisser correction (ε = .57) was employed in an ANOVA which did not show any significant effect of the degree of gaze-following on humanness ascription, *F*(3.4, 61.16)  = 1.11; *p* = .364, and hence confirms the initial observation. Repeated measures ANOVAs including experiment (experiment 3 vs. experiment 1; experiment 3 vs. experiment 2) as a between-subjects factor demonstrated that humanness ascription during competitive interaction differed significantly from cooperative interaction, *F*(3.31, 134.75)  = 14.17; *p*<.001, and showed a strong trend towards significance compared to the interaction with a naïve interactor, *F*(3.53, 148.21)  = 2.34; *p* = .056.

#### Discussion

As predicted, when interacting with a competitive interactor, neither congruency nor mere contingency of reactions played a role in influencing the ascription of humanness. This demonstrates that participants expect a competitive partner to avoid reciprocation and coordination, thus further corroborating the importance of congruency and contingency in experiencing an interaction as an interaction with a human interactor.

### Debriefing Questionnaires

For a better understanding of how participants addressed the task their responses in the post-experiment debriefing questionnaires were analyzed (see Figure S1). These questionnaires included four questions:

(1) Did participants base the ascription of humanness on considerations of human behavior or the function of a computer? Overall, the vast majority of participants based their ratings on considerations about human behavior (90.52%) rather than solely the function of computers (9.48%) while performing the task. This suggests that the non-verbal Turing test did not assess participant’s hypotheses about how computers are programmed but indeed the experience of interaction with other persons.

(2) How difficult did participants rate the task on a scale from 1 (easy) to 4 (difficult)? The condition to which participants were assigned had a significant effect on their difficulty ratings, *F*(2, 64)  = 6.04, *p* = .004. Tukey post-hoc comparisons of the three experiments revealed that difficulty ratings of participants who had interacted with a putatively cooperative interactor (i.e. Experiment 2) were significantly lower (M = 2.59, 95% CI [2.32, 2.88]) compared to difficulty ratings in the naïve (M = 3.1, 95% CI [2.86, 3.35]), *p* = .017, or competitive (M = 3.23, 95% CI [2.94, 3.51]), *p* = .004, condition. This indicates that the ascription of humanness was easiest for participants who had interacted with a cooperative interactor.

(3) Did participants use any behavioral strategy to unravel the nature of their interactor? An analysis of the presence of a strategy did not reveal any significant difference between the three conditions, *F*(2, 67)  = 1.84, p = .17, indicating that the nature of the interaction partner did not have any effect on how strategic participants addressed the Turing test.

(4) Could participants report any specific criterion for deciding between having interacted between a human and a computer? A one-way ANOVA revealed that the condition had a significant effect on whether participants had a specific criterion for humanness ascription, *F*(2, 67)  = 10.99, *p*<.001. Tukey post-hoc comparisons showed that participants who had interacted with a putatively competitive interactor had significantly fewer explicit criteria for humanness ascription (M = 0.10, 95% CI [0.01, 0.24]) compared to the naïve (M = 0.46, 95% CI [0.37, 0.78]), *p* = .032, or cooperative (M = 0.58, 95% CI [0.37, 0.78]), *p* = .003, condition. The proportion of explicit criteria did not differ between the naïve and the cooperative condition.

Eventually, we also looked at the comments in the questionnaires in a descriptive way. Notably, a considerable number of participants indicated that they expected a human interactor to either always follow their gaze or always avert their gaze and simply counted the occurrence of the expected reactions. In the following section two experiments including a concurrent cognitive load task will address the issue whether the Turing test provides a measure of strategic reasoning about humanness or rather of the implicit experience of an interaction as social.

### Experiment 4: Interaction with a Naïve Confederate under Increased Cognitive Load

The possibility that participants simply test ad hoc hypotheses about human behavior in order to solve the Turing test provides a potential problem to our approach which aims at unraveling the factors that lead to the phenomenological experience of an interaction as an interaction with another human rather than strategic behaviors that might inform a decision between mind and machine. Social cognition has been distinguished from other domains of cognition by a high degree of automaticity and reflexivity of its core processes [26], [27]. An increase of cognitive load in a so-called dual-task design is known to burden effortful *reflective* rather than automatic *reflexive* processes due to competition for limited cognitive resources [28]. In experiment 4 participants were instructed in the same way as in experiment 1. However, when the object changed color, a random number between 2 and 9 appeared superimposed on it. The concurrent cognitive load task consisted in adding up all six numbers that appeared during one interaction segment and to enter the sum after giving the response with respect to the nature of the interaction partner. We expected this manipulation to distract participants from any explicit strategy they could employ to inform the ascription of humanness.

#### Participants

26 volunteers participated in this experiment (M = 25.85, SD = 3.3; 14 female). One participant needed to be excluded from the analysis because he did not believe the cover story.

#### Results

**Figure 4:**
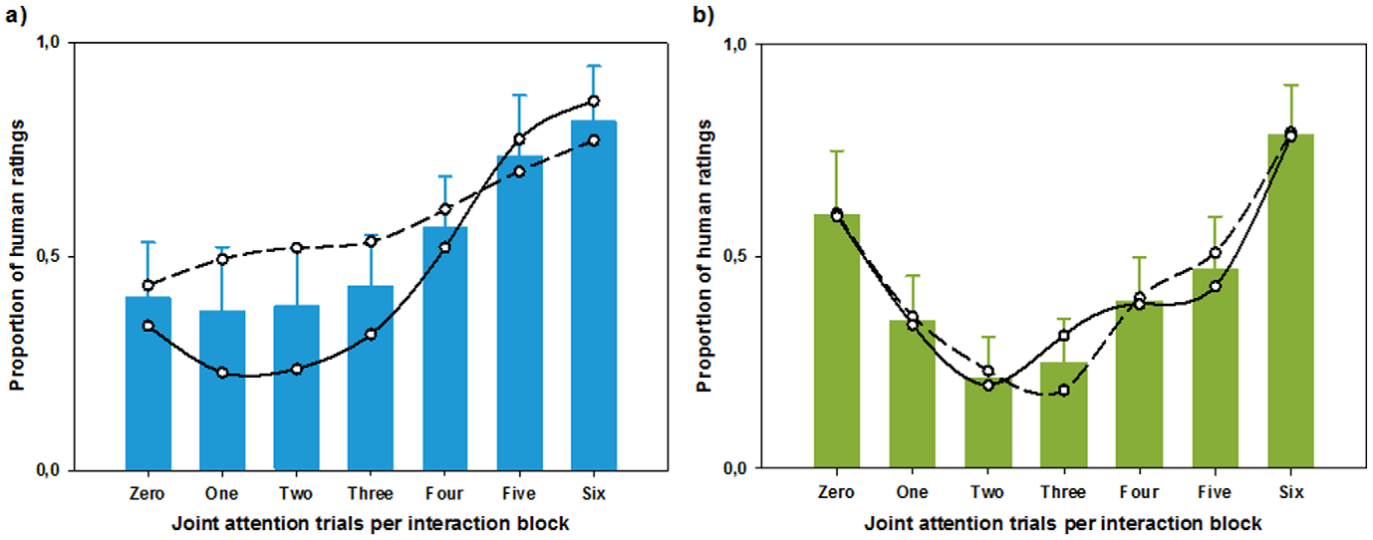
Experiments 4 and 5: The ascription of humanness to a virtual character while concurrently solving a cognitive load task. A median split separated participants with high and low scores in the cognitive load task. Solid lines represent the mean humanness ratings of high performers, whereas dashed lines represent low performers. *(a)* During naïve interaction cognitive load performance had an effect on humanness ascription (*p* = 0.49). High performers show a stronger congruency-based response pattern compared to low performers. *(b)* In cooperative interaction there was no effect of load performance on the ascription of humanness.

The results of humanness ascription during interaction with a naïve partner under cognitive load are depicted in Figure 4a. As in experiment 1, human ratings increase with increasing gaze-following. Mauchly’s test indicated that the assumption of sphericity was violated (χ^2^ = 90.23; *p*<.001) and degrees of freedom were corrected using the Greenhouse-Geisser estimates of sphericity (ε = .36). The results of a one-way repeated-measures ANOVA indicated a highly significant effect of gaze-following on humanness ascription, *F*(2.16, 47.51)  = 10.45, *p*<.001, ω^2^ = 0.24. Polynomial contrasts revealed both a highly significant linear, *F*(1, 20)  = 12.87, *p* = .001, ω^2^ = 0.29, and quadratic trend, *F*(1, 20)  = 11.09, *p* = .001, ω^2^ = 0.16, as in experiment 1. A repeated measures ANOVA including experiment as a between-subjects factor (experiment 4 vs. experiment 1) showed that humanness ascription during interaction with a naïve partner was not significantly affected by the presence of a concurrent cognitive load task, *F*(2.89, 137.51)  = 0.59, *p* = .62, and thus confirmed the results from experiment 1.

Participants of experiment 4 were eventually separated into a high- and a low-performance group by means of a median split based on cognitive load performance. A one-way repeated measures ANOVA with performance group as a between-subjects factor demonstrated a significant effect of cognitive load performance on the ascription of humanness, *F*(2.71, 56.86)  = 2.88, *p* = .049. Polynomial trend analysis within these two groups indicates that the high-performance group shows a stronger linear trend, *F*(1, 11)  = 21.9, *p* = .001, ω^2^ = 0.59, compared to the low-performance group, *F*(1, 11)  = 6.84, *p* = .024, ω^2^ = 0.28. This is illustrated by figure 4a which demonstrates that participants in the high-performance group (solid lines) show a much more pronounced congruency-based response pattern than those in the low-performance group.

#### Discussion

Overall, humanness ascription in the naïve condition did not change significantly under concurrent cognitive load. However, splitting participants into a low- and a high-performance group demonstrated a clear effect of the load manipulation: Participants who obtained higher scores in the load task showed a more pronounced linear trend in humanness ascription, that is, they based their ratings maximally on the congruency of the virtual character's reaction. As higher performance in the cognitive load task is indicative of greater distraction by this task, these results emphasize that the congruency of gaze-reactions is the most prominent cue for humanness ascription when cognitive resources are burdened during the Turing test. This can be taken to suggest that congruency-based responses are produced in an implicit and automatic fashion rather than being a product of strategic reasoning processes.

### Experiment 5: Interaction with a Cooperative Interaction Partner under Increased Cognitive Load

This experiment followed the same rationale as experiment 4 and assessed the effect of concurrent cognitive load on humanness ascription during interaction with a cooperative interactor.

#### Participants

In this experiment, 29 healthy volunteers participated (M = 25.11, SD = 4.42; 17 female). One male and a female participant were excluded from the analysis due to technical problems during the experiment.

#### Results

*Effect of Gaze Reactions.* As in experiment 3, the mean responses suggest that again overall contingency seems to play an important role in the experience of an interaction as social (Figure 4b). The Greenhouse-Geisser correction (ε = .47) was used to correct for the violation of sphericity as indicated by Mauchly’s test (χ^2^ = 84.24, *p*<.001). Again, the degree of gaze-following had a highly significant effect on humanness ascription, *F*(2.79, 72.64)  = 12.52, *p*<.001, ω^2^ = 0.29, and displayed significant linear, *F*(1, 26)  = 6.03; *p*<.021, ω^2^ = 0.05, and quadratic trends, *F*(1, 26)  = 25.42, *p*<.001, ω^2^ = 0.44. As indicated by a repeated measures ANOVA including the presence of the cognitive load task as a between-subjects factor (experiment 5 vs. experiment 2) the addition of a concurrent cognitive load task did not lead to group differences in humanness ascription, *F*(2.75, 134.57)  = 1.22, *p* = .31.

Participants again were separated into high- and low-performers by a median split of cognitive load performance. Unlike experiment 4 including load performance as a between-subjects factor did not yield any significant effect, *F*(2.65, 63.68)  = 0.36, *p* = .36. In contrast, humanness ascription differed significantly between the two cognitive load experiments (experiment 4 vs. experiment 5), *F*(3.12, 146,47)  = 3.81, *p* = .011, thus indicating that the difference in response patterns observed in naïve compared to cooperative interactions remained consistent despite the addition of a cognitive load task.

#### Discussion

The results of this experiment confirmed that humanness is ascribed based on the mere contingency of gaze reactions when the Turing test is performed with a cooperative interactor. Both high- and low-performers equally ascribed humanness based on contingent rather than congruent responses, indicating that contingency is the prevalent cue irrespective of the degree of cognitive burdening imposed by the cognitive load task. The cooperative interaction hence seems to induce an implicit expectation of contingency that is not altered by any strategic reasoning.

### Further Hints to the Implicitness of Humanness Ascription

**Figure 5:**
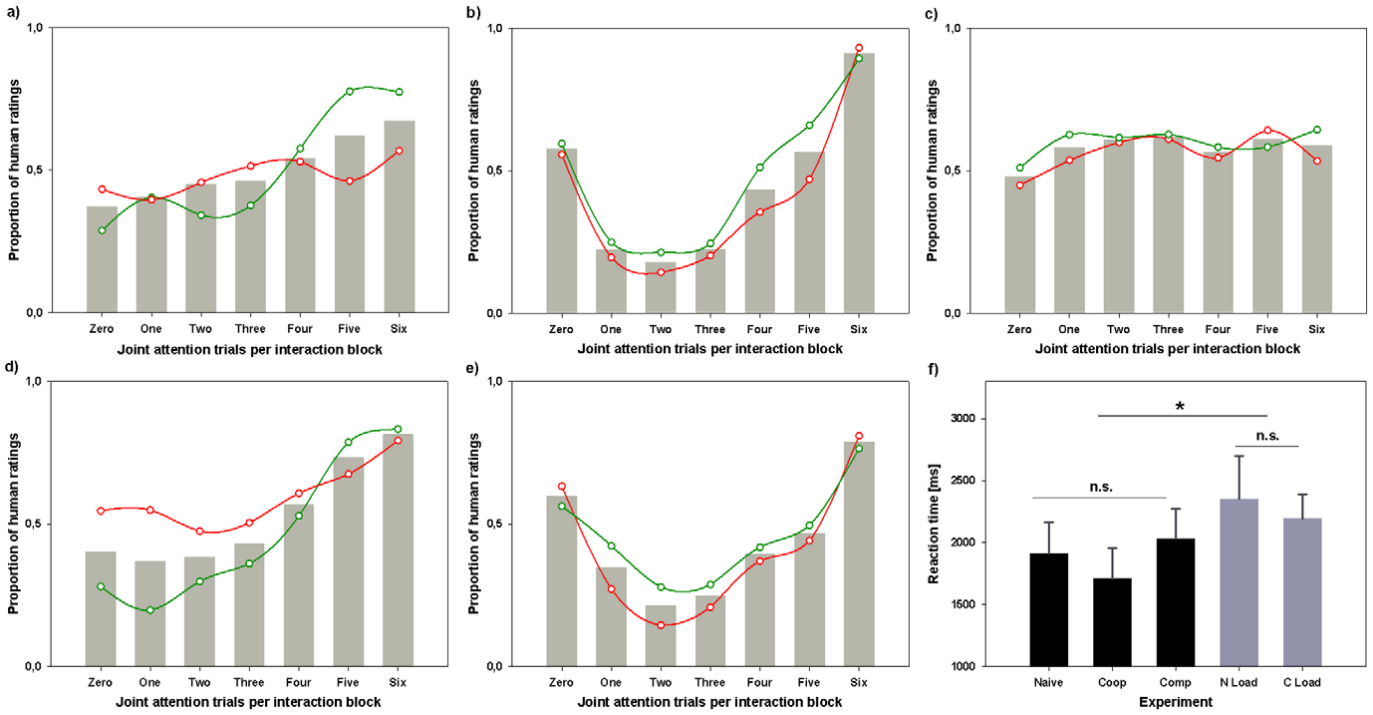
Reaction times of humanness decisions split by median. Grey bars indicate mean ratings. Mean ratings of fast responders (reaction time below median) are indicated by green scatter plot, mean ratings of slow responders are indicated by red scatter plot. Effects of response time are indicated in brackets. *(a)* Naïve interactor (*p* = .034): In fast responders humanness ascription is driven more strongly by congruency than in slow responders. *(b)* Cooperative interactor (n.s.). *(c)* Competitive interactor (n.s.). *(d)* Naïve interactor + cognitive load (*p* = .004): Fast responders show stronger congruency-based response patterns compared to slow responders. *(e)* Cooperative interactor + cognitive Load (n.s.). *(f)* Mean reaction times for all experiments (see text for details).

In the two cognitive load experiments the focus of the manipulation was during the interaction phase. The rationale was that the task would distract people from thinking about the interaction process and engaging in strategic reasoning about the task. Nevertheless, the decision between human or computer might not emerge *during* but completely *after* the interaction. To address this possibility we analyzed reaction times (see Figure 5d). A one-way ANOVA including all experiments was performed and demonstrated a main effect of experimental group on reaction times, *F*(4, 116)  = 3,79, p = .006. Pooling the data into load and no-load experiments showed that this effect was due to significantly higher reaction times in the load (M = 2250.7, SE = 94.4) compared to the no-load (M = 1877.81, SE = 70.23) tasks, t(119)  = −2,56, *p* = .012, suggesting higher cognitive demands resulting from the combination of the humanness ascription and the cognitive load task. A one-way ANOVA did not reveal any significant differences of reaction times between the no-load conditions (experiments 1, 2, and 3), *F*(2, 68)  = 2.01, *p* = .142. A comparison of the two load experiments (experiments 4 and 5) also did not show any significant difference, t(48)  = .92, *p* = .364. Although this suggests that the decision is made during the interaction, it cannot be ruled out that reasoning processes between the end of the interaction block and the button press play a role in humanness ascription.

To investigate this matter, a median split of reaction times was performed for all experiments (Figure 5a–e). In the naïve condition, a one-way repeated-measures ANOVA revealed a significant effect of reaction time on humanness ascription, *F*(3.09, 70.99)  = 3.02, *p* = .034, ω^2^ = 0.12. Separate ANOVAs for participants with short and long reaction times showed that the degree of gaze-following only had an effect in the fast responders, *F*(2.82, 33.79)  = 5.39, *p* = .004, ω^2^ = 0.32, who showed a highly significant linear trend of humanness ascription, *F*(1, 12)  = 13.02, *p* = .004, ω^2^ = 0.32. In the slow responders, there was no such effect, *F*(3.4, 37.36)  = 1.23, *p* = .31. In the naïve condition including cognitive load an ANOVA revealed an effect of reaction time on the ascription of humanness, *F*(2.52, 55.48)  = 5.44, *p* = .004, ω^2^ = 0.22. Similar results as in the naïve condition without cognitive load were indicated by separate ANOVAs for slow and fast responders. Gaze-following only had a significant effect in the fast responders, *F*(1.74, 20.83)  = 17.13, p<.001, ω^2^ = 0.44, where also a linear trend was present, *F*(1, 12)  = 24.04, p<.001, ω^2^ = 0.43, but not in the slow responders, *F*(3.36, 33.55)  = 1.8, p = .11. This suggests that the longer participants think about their decision after the interaction, the lesser they take congruency into account as a humanness cue. Interestingly, there was no such an effect for experiments 2, 3, and 5, indicating that during cooperative interaction, the ascription of humanness is implicitly based on the contingency of gaze reactions without being influenced by the time spent on thinking about the decision.

The reaction time data are supported by participant's responses to the questions whether they had behavioral strategies and whether they could mention explicit criteria for humanness ascription. Concerning the question whether they had used specific strategies to investigate whether they had interacted with another human or a computer (Figure S1c), this was significantly less the case in the experiments including a concurrent cognitive load task, χ^2^(1)  = 6.23, *p* = .013. In addition, although this was only a statistical trend, participants did report specific criteria for the ascription of humanness (Figure S1d) considerably less often in the cognitive load experiments compared to the experiments with increased cognitive load, χ^2^(1)  = 3.27, *p* = .07. These results indicate that manipulation of cognitive load was successful in reducing strategic behavior of participants as well as their awareness of specific criteria for the ascription of humanness.

## Discussion

In a series of experiments, we have made use of a novel interactive eye-tracking paradigm to establish what we describe as a non-verbal Turing test. This setup makes it possible to assess parameters of gaze-based interaction which lead to the experience of a truly social encounter with a real human interaction partner. Hereby we could overcome the paradoxical situation of previous studies on social cognition in which the behavior of a single person is observed in isolation from others. The experience of being involved in interaction is constituted by two aspects: Firstly, participants in our experiments experience that they are directly addressed by the virtual character whose gaze behavior is made contingent on their own in real time. The necessity of “being addressed as you” has recently been advanced as a second-person approach to social cognition in the fields of social cognition and neuroscience [5], [29], [30]. Secondly, the paradigm enables participants to directly observe the consequences of their actions on another agent as it would occur in real-life interaction. This is vital for making sense of one’s own behavior in an interactive context and for its adjustment to situational requirements.

This newly developed approach provides important and novel insights on the process underlying the ascription of humanness to virtual characters in social encounters. In order to model different social contexts, participants engaged in the non-verbal Turing test under changing situational demands: Experiment 1 assessed humanness ascription during interaction with an interactor who was thought to be *naïve* to the task in order to assess the default ascription pattern when there is no knowledge about the interactor. Consistent with our hypothesis, participants activated a congruency-based expectation and increasingly ascribed humanness to the virtual character with increasing degrees of gaze-following. The results of experiment 2 demonstrate that this pattern can be modulated depending on the previous knowledge about the behavioral predisposition of the interaction partner and changes to a contingency-based analysis of behavior in the presence of a *cooperative* partner. As predicted, experiment 3 showed that the ascription of humanness during interaction with a *competitive* interactor was neither based on congruency nor on contingency of gaze reactions.

### The Special Case of Gaze

Before turning to an in-depth discussion of our results there are two controversial issues related to the operationalization of the interaction process using gaze cues and to the resulting explanations that need to be addressed.

First of all, it might be argued that gaze-following is merely a form of motor mimicry which refers to a subtle imitation of the behavior of an interaction partner. Consequently, the ascription of humanness might rely on mimicry-related processes which are known to increase rapport, empathy, and liking between mimicker and mimickee and thereby result in increased bonding of the interactors [31]. Although gaze-following naturally has an imitative component, motor mimicry can clearly be distinguished from gaze-following in a number of respects. Chartrand and Bargh [32] describe mimicry as non-conscious imitation “such that one’s behavior passively and unintentionally changes to match that of others in one’s current social environment” (p.893). The involvement of a distinct task as in our series of experiments makes it difficult to argue that the other’s gaze-following is passive or unintentional. Another important argument is that the appraisal of the mimicker decreases and other positive social effects break down once the mimickee becomes aware of being mimicked, possibly because the imitative behavior is evaluated as an intentional expression of conformity directed at the attainment of reward or approval [33], [34]. In our task it is obvious that the participant is aware of the other following or not following his gaze, as this is the criterion on which the decision between human and computer is based. A further distinction concerns the *function* of mimicry and gaze-following. Whereas the main function of mimicry seems to be the general facilitation of dyadic interactions, gaze-following is related to triadic rather than dyadic interactions where it serves the purpose of keeping track of another person’s focus of attention, thereby paving the way to an understanding of this person’s mental states [9], [10]. The distinct task structure, participants’ awareness of the other’s reactions, as well as the functional role of gaze-following clearly argue against any substantial role of mimicry in the present study.

Secondly, a critical reader might ask whether the effects demonstrated here are gaze-specific or whether they could potentially be replicated using different channels of non-verbal behavior. Undoubtedly, some of the effects reported in this article might appear in a Turing-test-like study involving other forms of interaction. However, the aim of the present study was to uncover the basic aspects of ‘online’ social interaction that lead to the experience of this interaction as an interaction with another human. In order to obtain a valid operationalization, we identified and aimed at fulfilling two main criteria without which the task could not provide a valid experimental investigation of online social interaction. First, the task needs to provide a high level of ecological validity, i.e. both channel and process of the interaction must be highly salient in everyday social interactions. Second, the task must provide a high degree of experimental control. Obviously, other cue system could be used to model contingency and congruency of an interaction in an experimentally controllable fashion. For example, a similar study design could involve pressing a button, moving a cursor, producing a sound or any combination of these cues. However, this would not satisfy the criterion of ecological validity as these activities are not part of every-day social interactions. Furthermore, social gaze is distinct from other communicative channels in one crucial aspect. Already more than 40 years ago, Gibson and Pick noted that gaze “can be treated as a source of stimulation as well as a type of response. The eyes not only look but are looked at” ([13], p.386) and that hence in the act of looking perception and action are inseparable. Taken together, for the following reasons, social gaze seems most ideally suited for a Turing-test-like assessment of social interaction: (i) It readily occurs in natural interaction, (ii) it is linked to an understanding of other’s minds, (iii) it is easily controllable in an experimental setting, and (iv) it combines stimulation and response in one action.

### The Valence of Gaze Aversion and Gaze-Following

As a key finding, our studies demonstrate that human beings who interacted with a putatively naïve partner displayed an implicit expectation of gaze-following behavior and experienced an interaction as social when the interactor followed their gaze and engaged in joint attention with them.. This effect is surprisingly robust given that the only piece of information available to the participants was that the partner had been instructed to react to their gaze by “freely choosing to look to the same or the other object” without being able to willingly help them to solve the task.

*Why is maximally averted gaze not indicative of a human interactor?* In the first instance, this might be related to the fact that in the domain of social gaze valence is an inherent property of the contingency continuum which ranges from maximal gaze aversion to maximal gaze-following [6]. The neglect of maximal gaze aversion as a cue to humanness during interaction with a naïve interactor might be related to the negative valence of gaze aversion that has been demonstrated on various levels. For example, in a study on the effects of gaze cues on person construal it has been shown that participants produced higher ratings of both likeability and attractiveness for pictures of people shifting the gaze towards them compared to pictures of people averting their gaze from them [35]. In another study [36], participants viewed video sequences displaying a human face either directing its gaze at them or averting it by looking left or right from time to time. As a between-subjects factor the degree of gaze aversion was varied. After having watched the movies, participants had to fill out a social rejection questionnaire which showed that feelings of exclusion and ostracism increased with increasing total duration of gaze aversion. In addition, gaze aversion generally increased feelings of negative mood and decreased prosocial attitudes. Additional evidence for the negative valence of gaze aversion comes from an EEG experiment in which participants viewed live faces displaying either direct or averted gaze [36]. An analysis of EEG activity revealed that direct gaze elicited left-hemispheric frontal activation which has been related to approach motivation. On the contrary, averted gaze resulted in right-sided frontal activation that has been related to an avoidance motivation, suggesting that gaze aversion triggers neural responses related to negative affect [37].

*Is there comparable support for a positive valence of gaze-following and joint attention?* A crucial distinction has been made between other- and self-initiated joint attention. One can either respond to bids of joint attention by others or initiate joint attention by leading someone’s gaze. Whereas gaze-following has been observed in other species, the ability and spontaneous motivation to lead someone’s gaze is uniquely human. Its function is to share interests and pleasant experiences regarding objects in the environment with others [10]. For the present study, a recently discovered motivational aspect of self-initiated joint attention is of great importance. Schilbach and colleagues [16] report that being involved in joint attention, irrespective of its initiator, results in the activation of regions of the so-called “social brain”, such as the medial prefrontal cortex. This region has been implicated in mentalizing, i.e. in thinking about other person’s goals and intentions [38]. Initiating joint attention oneself, however, is associated with increased neural activity in the ventral striatum as part of the brain’s reward system whose activity changes have been linked to hedonic experiences and the anticipation of reward [39], [40]. In addition, there was a significant correlation of the strength of striatal activation with ratings of the pleasantness of joint attention obtained in a post-scan questionnaire. These findings indicate that self-initiated joint attention triggers reward-related processing and hence provides an intrinsic motivation for engaging others in joint attention. In other words, we seek for reciprocation and enjoy being able to elicit congruent responses from others to our actions. Taken together, we believe that these positive connotations of gaze-following may be crucial in informing the ascription of humanness.

### From Joint Attention to Joint Action by Cooperation

Our results provide compelling evidence for the significant impact of prior knowledge about the goal of the presumed interactor on the experience of an interaction. When the interactor was explicitly introduced as cooperative, the ascription of humanness was not based on congruency but rather followed the actual contingency of the virtual character’s reactions more closely. This finding is consistent with our hypothesis and indicates that people were, in fact, not blind to the actual contingencies, but only integrate them when the interactor’s disposition to cooperate is known.

*How can cooperation lead to the discounting of the expectation of congruent gaze reactions?* Cooperation, in the traditional view, is a behavior that is selected to provide mutual benefit to both the actor and the recipient. Cooperation often requires that immediate benefits are discounted in order to gain a delayed reward [41], [42]. However, cooperation has not only been defined in terms of its fitness consequences, but also in a mechanistic sense as a form of behavioral coordination [18]. In this definition, particular emphasis is put on the necessity of coordination between the cooperative interaction partners which is regarded as an “important proximate mechanism needed to accomplish cooperation” ([43], p. 7). Interestingly, the coordination of behaviors is not only pivotal for cooperation, but also for joint action [44]. For example, musicians playing instruments in a band, a couple dancing together, or construction workers building a house demonstrate cases of joint action. It is hence possible that the discounting of mere congruency in the cooperative condition is a consequence of participants interpreting the interaction as a form of joint action. An analysis of the degree of coordination expected by participants from a human interactor and an assessment of the criteria that an interaction needs to fulfill in order to be classified as a joint action might help to assess this option.

*Does cooperative interaction in the non-verbal Turing test qualify as a joint action?* There are two salient coordinated behavioral patterns that occur in the Turing test, namely maximal gaze-following or maximal gaze aversion. Data from the naïve condition suggest that maximal gaze-following constitutes the most basic and effortless form of coordinative behavior which seems to be expected “by default” when people engage in interaction. In the cooperative situation, any form of contingency is judged as indicative of a human interaction partner, thus indicating that participants expect a higher degree of coordination. This strong expectation of coordinated behavior irrespective of the congruency of reactions might be taken to suggest that participants understand the cooperative interaction as a situation of joint action. Fiebich and Gallagher [45] have recently identified three conditions that need to be satisfied before interactors can be said to be engaged in joint action: i) they need to have a shared goal or intention, ii) they must have common knowledge of aiming at this goal together, and iii) they have to participate in coordinated patterns in order to reach this goal. These criteria are fulfilled in the cooperative version of the Turing test: (i) The shared goal of increasing the common monetary reward is easily identified for the interaction with a cooperative interaction partner. (ii) As this has been communicated explicitly, the participant can also assume that they are aiming at this goal together. (iii) The contingency-driven response pattern indicates that participants strongly expected the other to coordinate his behavior to their actions on a higher level than mere congruency. We speculate that this demonstrates an intrinsic expectation of higher-order coordination in cooperation compared to the unrestrained interaction format in the naïve condition and thus provides evidence that the interaction with a cooperative interactor is automatically interpreted as a situation of joint action.

#### Experiencing Interaction or Thinking about Interaction?

It might be argued that the ascription of humanness could have been based on reasoning processes which are not related to the experience of social interaction. Social cognition has been described as being largely constituted by automatic processes are fast, unconscious, and do not require willful regulatory efforts [27], [46]. Hence, if participant’s judgments were the outcome of conscious, deliberate, and strategic thought processes this would pose a problem to our claim of presenting these judgments as measures of the experience of interacting with another human. We assessed this possibility in several respects. First of all, the addition of a concurrent cognitive load task in experiments 4 and 5 specifically aimed at interfering with strategic processes during the interaction process by burdening the cognitive system of participants.

The results of these experiments clearly demonstrated that during naïve interaction the increase of cognitive load lead to an increased in congruency-based humanness ascription. Notably, participants who obtained high scores in the cognitive load task based the ascription of humanness more strongly on congruency than participants with low scores. This indicates that the interference created by the load task unraveled implicit or automatic response patterns. In cooperative interaction, on the other hand, the presence of the cognitive load task had no effect on humanness ascription, demonstrating that contingency-based responses represent implicit judgments of humanness. Overall, participants in the experiments including cognitive load reported that they used less strategies and less explicit criteria of humanness ascription, thereby further corroborating the effectiveness of the load manipulation. Considering that the decision between human and computer could take place completely after the interaction itself, reaction times were analyzed by splitting participants into fast and slow responders. In interactions with a naïve interactor, irrespective of the presence of a cognitive load task, fast responders base humanness ascription more strongly on congruency than slow responders. Taken together, these findings indicate that we were able to address the implicit processes leading to the experience of an interaction as an interaction with a human agent rather than results of careful deliberation that might inform a decision between mind and machine.

### Outlook and Conclusions

Insights into how congruency and contingency of reactions to our own gaze behavior lead to the experience of an interaction as social address the interests of various fields of research. For instance, the current paradigm is likely to provide a useful tool to investigate impairments of the ability to engage in online social interaction in psychiatric disorders, such as it is observed in schizophrenia and autism [2]. The current methodological developments and empirical results could also inform research on human-computer interfaces aiming at the development of virtual agents that appear and behave human in a natural way in order to facilitate smooth interaction [47], [48]. Clearly, such developments can benefit from research unraveling the core aspects of human social interaction by using truly interactive paradigms. Most obviously, however, the adaptation of the present experimental design for neuroimaging studies will provide a powerful tool for the study of the neural underpinnings of social interaction. In this respect, it can be hypothesized that gaze-based interaction with a naïve confederate might lead to an increase in neural activity in areas of the mentalizing system such as the medial prefrontal cortex [49]. In addition, conditions with highly congruent reactions might correlate with increased activity in brain areas implicated in the processing of reward such as the amygdala and the ventral striatum [16], [50], [51]. While competitive interaction might also concur with an increase of neural activity in mentalizing areas it would be interesting to investigate whether the competitive context could also lead to a decrease of activity in reward-related neurocircuitry when observing joint attention. Likewise, an interesting question concerns the neural substrates of contingency evaluation in a cooperative context: Does the presence of a shared goal lead to a decrease of activity in the mentalizing system in favor of activation of brain areas implicated in coordinated behavior (e.g., [52])? Furthermore, it will be interesting to investigate whether changes in activation of the reward system in response to positively contingent gaze-reactions could generalize to contingent reactions irrespective of their valence depending on the situational context.

In summary, our results demonstrate that the use of innovative methodology and experimental designs makes it possible to address the interaction process itself instead of focusing on the study of single minds in isolation [1]. Though still rare, truly interactive paradigms have also been advanced by other researchers in psychology and cognitive neuroscience [1], [53]–[56]. This emphasizes the need for such studies if we want to understand why and how we interact with others in a more sophisticated way than any other species.

## Supporting Information

Figure S1 Overview of participants' responses to the post-experiment debriefing questionnaire.
(TIF)
