# Supplementary figures and images for: A Non-Verbal Turing Test: Differentiating Mind from Machine in Gaze-Based Social Interaction
Source: PLoS One. 2011 Nov 9;6(11):e27591. doi: 10.1371/journal.pone.0027591 (PMC3212571; doi:10.1371/journal.pone.0027591)

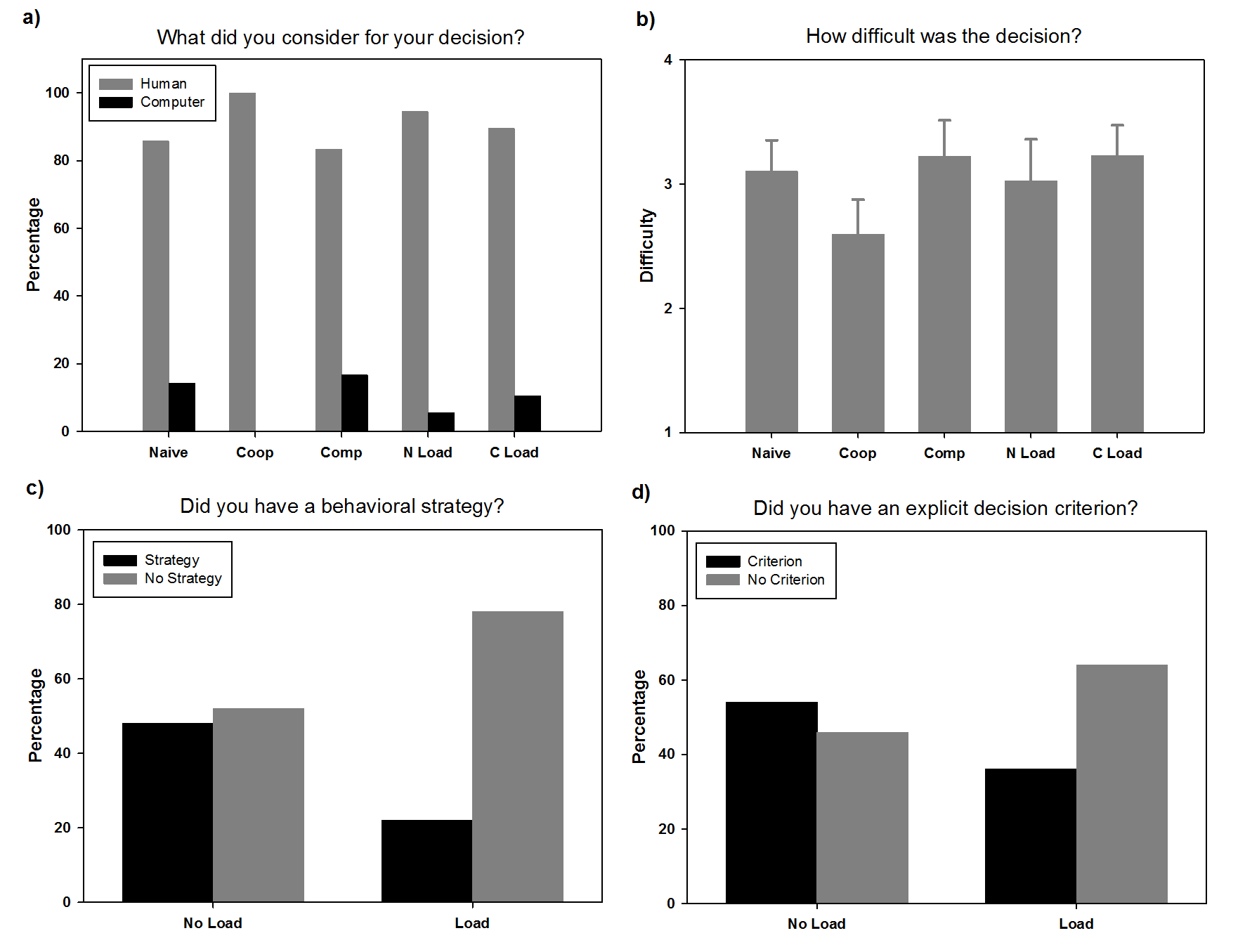

Supplement: Figure S1 — Overview of participants' responses to the post-experiment debriefing questionnaire. (TIF) [file pone.0027591.s001.tif]
